# Extra-Curricular Activities Improved Reproductive Health Knowledge of Ethnic Minority High School Students in Vietnam 

**Published:** 2019-06

**Authors:** Mai Van Hung, Duong Van Khoa, Hoang Quy Tinh, Trinh Hai Thuy, Nguyen Phuc Hung

**Affiliations:** 1 Department of Science Education, Center for Anthropology and Mind Development, Department of Science Education, VNU University of Education, Hanoi, Vietnam; 2 Faculty of Politic Theory - Civic Education, Hanoi National University of Education, Hanoi, Vietnam; 3 Faculty of Early Childhood Education, Hanoi National University of Education, Hanoi, Vietnam; 4 Ethnic Minority High School, Dien Bien Dong District, Dienbien Province, Vietnam; 5 Centre for Reproductive Health Education and Family Planning, Faculty of Biology, Hanoi National University of Education, Hanoi, Vietnam

**Keywords:** Reproductive Health, Ethnic Minority, High School, Extra-Curricular Activity

## Abstract

**Objective:** To assess the effectiveness of extra-curricular activities in improving reproductive health knowledge of ethnic minority students in mountainous areas in Vietnam.

**Materials and methods:** The study was conducted on 400 ethnic minority students at Dien Bien Dong Ethnic Minority High School in Vietnam. The selected healthy students with a similar mix of study results and grades were divided into two groups: a control group had no the extra-curricular activities on reproductive health, and an experimental group participated extra-curricular activities on reproductive health. The extra-curricular activities were designed as a series of seminars on numerous reproductive health contents. The retention of reproductive health knowledge was then evaluated by a test containing multiple-choice and single-choice questions.

**Results:** Results showed that the percentage of students who did not correctly understand puberty signs, ovulation time during menstrual cycle, contraceptive method use and sexually transmitted diseases in the control group ranged from 34.5% - 83.5%. Despite the fact that the ethnic minority high school students’ knowledge of reproductive health was poor, the percentage of students who fully understood puberty signs, ovulation time during menstrual cycle, contraceptive method use and sexually transmitted diseases significantly increased and reached at least 90% after attending extra-curricular activities on reproductive health (p < 0.05).

**Conclusion:** The ethnic minority high school students’ knowledge of reproductive health was relatively poor. Extra-curricular activities markedly increased the knowledge of many reproductive health aspects. These findings suggest that it is necessary to improve the knowledge of reproductive health for ethnic minority high school students in mountainous areas in Vietnam, and that extra-curricular activities organized as seminars are effective and suitable to provide and retain students’ knowledge of reproductive health.

## Introduction

The age of adolescence is an important stage in the development of the body, characterized by major changes of both psychological and physiological aspects (1). During this period, adolescents are affected by numerous factors including individual, family, community and society. The minors tend to become more precocious at the age of puberty, and the age which they have the first sexual intercourse has been falling. However, most adolescents do not have adequate knowledge of reproductive health (2, 3, 4). This is an important reason leading to an increase in premarital sex, unwanted pregnancies, abortion rates and the spread of sexually transmitted diseases, including HIV/AIDS. With the reported number of 300,000 abortions per year and 20% of those involving in adolescents, Vietnam has the highest abortion rate of adolescents in Southeast Asia and ranks fifth in the world (3, 5, 6). Moreover, it has been reported that there were about 800.000 to 1.000.000 people infected with a sexually transmitted disease each year and the adolescents accounted for 40% of these people (6, 7).

In recent years, reproductive health education in Vietnamese high schools is said to be carried out as an method called “integration of disciplines”. However, it has been shown that this method is not effective (8, 9). There are some reasons for the ineffectiveness of the integration of disciplines, such as: the little integration of reproductive health contents in other subjects, no obligation on the side of the teachers to include reproductive health education as a part of the curriculum, and the uncomfortableness to share about reproductive health aspects due to the traditional culture. Therefore, it is essential to seek other methods which would improve students**’** knowledge of reproductive health aspects through increasing their attention. In previous study, we have found that extra-curricular activities markedly improved reproductive health awareness of high school students in urban areas (9). However, the effectiveness of this method on those in mountainous has not been examined yet. In addition, high school students in mountainous areas mostly belong to ethnic minority people and they are considered to have poor knowledge in general. Hence, the aim of this study was to evaluate the necessity and effectiveness of the extra-curricular activities to improve the knowledge of ethnic minority high school students on reproductive health.

## Materials and methods

***Study design:*** A study population consisting of 400 healthy ethnic minority students at Ethnic Minority High School in Dien Bien Dong district, Dien Bien province, Vietnam was chosen. The students and their parents were asked for an agreement to participate the study. Then these students were randomly divided into two groups: the control group and the experimental group (200 students per group), ensuring a similar mix of study results and grades of students in each group. The control group took part in no extra-curricular reproductive health activities. The experimental group participated in extra-curricular reproductive health activities. The extra-curricular activities were conducted outside the regular classes during school hours. Six months after extra-curricular activities, all students were given a written test to evaluate the their knowledge of reproductive health.

***Extra-curricular activity:*** The extra-curricular activities in this study were a series of three specialized seminars designed by Centre for Reproductive Health Education and Family Planning, Hanoi National University of Education. The seminars provided the knowledge of reproductive health contents for adolescents, including: anatomy and physiology of reproductive system, puberty stage, conception and contraceptive methods, abortion, sexual diseases and sexually transmitted diseases. Each seminar was organized once a week and lasted for 45 minutes. This time period of the seminar was similar to a normal class hour at high schools. Total 200 students in the experimental groups were divided into 5 classes (40 students in each class). They were given one short lecture of reproductive health for 15 minutes. The lecture included main issues of reproductive health using videos, pictures and real situations relating to reproductive health in order to increase students’ attention. Then the students in each class were divided into small groups (5 students per group) and they had 15 minutes to discuss with group members about educative situations given by the lecturers. Finally, the representatives of each group provided their own knowledge and opinions following evaluations and conclusions of the lecturers within the last 15 minutes. 

***Data collection and analysis:*** In this study, the retention of reproductive health knowledge was evaluated after 6 months conducting extra-curricular activities by a test given to each student in both groups.

**Table 1:** Knowledge of puberty signs

**Puberty signs**	**Control group**	**Experimental group**	**P-value**
Increase in body length and weight	85.0	93.5	0.0417
Growth of pubic hair	88.5	97.0	0.0315
Change in character	77.5	96.5	0.0217
Attraction of gender	72.0	92.5	0.0186
Appearance of acne	89.0	98.5	0.0289
First appearance of menstruation in female	65.5	93.5	0.0126
First appearance of ejaculation in male	70.0	94.0	0.0135

Data were from multiple-choice questions (n = 200) and expressed as frequency percentage; p < 0.05 was level of significance.

The test containing numerous multiple-choice and single-choice questions of different reproductive health issues was prepared and approved by Centre for Reproductive Health Education and Family Planning, Hanoi National University of Education. For accurate evaluation of knowledge, participants could only choose “True” or “False” for their answers and they were required to finish all questions in the test. Also, students were not allowed to use any references and to discuss with others during the test. The completed questionnaires were assessed and analyzed using the Statistical Package for the Social Sciences (SPSS) software for Windows version 16.0. The significance is based on a 5% level of probability.

## Results

***Knowledge of puberty signs:*** Table 1 shows that the percentage of control students answering correctly different puberty signs ranged from 65.5% to 89.0%. These mean relatively high number of students did not know fully all given puberty signs (11.0% to 34.5%). In addition, the lowest percentage of students who had correct answers for the first appearance of menstruation in female (65.5%) and the first appearance of ejaculation in male (70.0%) was observed in control group though these are two important puberty signs. These results indicate that the knowledge of ethnic minority high school students on puberty signs was relatively poor. In contrast, the knowledge of all puberty signs of students in the experimental group increased significantly (p < 0.05). The percentage of students answering correctly signs of puberty was at least 93% (ranging from 93.5% to 98.5%). Moreover, the number of students who knew the two important above-mentioned signs markedly increased by extra-curricular activities (Table 1).

***Knowledge of ovulation time during menstrual cycle:*** Students’ knowledge of period of time during menstrual cycle in which the ovulation is most likely to occur was relatively poor in the control group (Table 2). Only one-third students (32%) in this group chose an appropriate answer for this knowledge while wrong answers were found up to 27.5%. However, the students’ knowledge on this reproductive health content dramatically improved in the control group (p < 0.05). After attending seminars, the percentage of students with an correct answer was elevated up to 72.0% and those of students with wrong answers were down to 8.0%.

**Table 2:** Knowledge of ovulation time during menstrual cycle

**Ovulation time **	**Control ** **group**	**Experimental ** **group**	**P-value**
Day 1 to day 7	13.5	2.5	0.0062
Day 8 to day 11	21.0	9.5	0.0341
Day 12 to day 18	32.0	72.0	0.0115
Day 19 to day 22	19.5	10.5	0.0425
Day 23 to the end	14.0	5.5	0.0156

Data were from single-choice questions (n = 200) and expressed as frequency percentage; p < 0.05 was level of significance.

***Knowledge of contraceptive method use:*** Students’ knowledge of contraceptive method use is shown in Table 3. In the control group, the percentage of students who knew three common contraceptive methods (condom, oral contraceptive pill and intra-uterine device) was relatively high (84.5%, 81.5% and 72%). However, the students’ knowledge of other contraceptive methods was poor (ranging from 12.0% to 52.0%). By participating extra-curricular activities, the knowledge of contraceptive method use was significantly improved (p < 0.05). Almost students in the experimental group knew all the given contraceptive methods (at least 90%) (Table 3).

***Knowledge of sexually transmitted diseases:*** Table 4 shows that students in the control group had relatively poor knowledge of sexually transmitted diseases, especially genital herpes (16.5%) and condylomaacuminata (23.0%). After attending the extra-curricular activities on reproductive health, the knowledge on sexually transmitted diseases markedly improved in the experimental group. The percentage of students who knew all five given diseases was greater than that of the control group (at least 90%). The differences in awareness of all diseases between the control group and the experimental group were statistically significant (p < 0.05). These results indicate that the extra-curricular activities had high effectiveness in improving students’ knowledge of sexually transmitted diseases.

**Table 3:** Knowledge of contraceptive method use

**Methods**	**Control ** **group**		**Experimental ** **group**		**P-** **value**
Calendar rhythm	25.5		91.0	0.0097	
Coitus interrupts	41.0		94.5	0.0102	
Condom	84.5		99.5	0.0208	
Oral contraceptive pill	81.5		96.5	0.0239	
Contraceptive implant	12.0		87.5	0.0075	
Contraceptive patch	14.5		89.5	0.0084	
Intra-uterine device	72.0		97.0	0.0143	
Tubal ligation and vasectomy		52.0	91.5	0.0116	

Data were from multiple-choice questions (n = 200) and expressed as frequency percentage; p < 0.05 was level of significance.

## Discussion

The knowledge of reproductive system, puberty, contraceptive methods and sexually transmitted diseases is important for adolescents since sexual intercourse is said to be more common in this age stage (3, 4, 6). Therefore, it is necessary to provide reproductive health knowledge for adolescents including high school students. In this study, the ethnic minority high school students’ knowledge of different reproductive health contents was relatively poor. 

**Table 4:** Knowledge of sexually transmitted diseases

**Diseases**	**Control ** **group**	**Experimental ** **group**	**P-value**
Gonorrhea	45.5	93.0	0.0153
Syphilis	48.0	96.5	0.0164
HIV/AIDS	85.5	99.5	0.0327
Genital herpes	16.5	90.5	0.0088
Condylomaacuminata	23.0	92.0	0.0094

Data were from multiple-choice questions (n = 200) and expressed as frequency percentage; p < 0.05 was level of significance.

There was high percentage of students who did not correctly understand puberty signs (11% - 34.5%, Table 1), ovulation time during menstrual cycle (68%, Table 2), contraceptive method use (12.0% - 52.0%, Table 3) and sexually transmitted diseases (14.5% - 83.5%, Table 4). It has been reported that high school students’ knowledge of reproductive health in urban areas in Vietnam was poor when a great number of students (accounting for 25% - 40%) had incorrect answers for reproductive health questions (9, 10, 11, 12). However, the knowledge of students revealed in those studies was still higher than that found in the present research. Ethnic minority students living in mountainous areas have few chances to gain reproductive health knowledge from different sources due to poverty, low living standards and geographical transporting difficulties. Hence, these may be the reasons responsible for the poorer knowledge of high school students found in our study.

Reproductive health education in Vietnamese high schools has been carried out as an method called “integration of disciplines” in recent years. However, the poor knowledge of reproductive health in the present study indicates that the reproductive health education which is now integrated into school curricular subjects, such as Biology, Geography and Citizen Education, has low effectiveness to provide and improve reproductive health knowledge for students. Also, this result suggests that it is highly required to implement extra-curricular education activities to increase reproductive health knowledge for high school students, especially ethnic minority ones in mountainous areas.

Despite the fact that the ethnic minority high school students’ knowledge of reproductive health was poor, it was markedly improved by attending extra-curricular activities. The percentage of students fully understanding puberty signs, ovulation time during menstrual cycle, contraceptive method use and sexually transmitted diseases increased significantly and reached at least 90% (p < 0.05). In particular, the knowledge of some difficult reproductive health contents (ovulation time during menstrual cycle and sexually transmitted diseases) which was the poorest in the control group had been improved dramatically. In the present study, students in both control and experimental groups were selected at the same high school and were ensured to have similar study results and grades, therefore, the better knowledge found in the latter group compared to that in the former group prove extra-curricular activities through seminars had high effectiveness to improve and retain knowledge of high school students on reproductive health. Moreover, each seminar was designed to fit with a class hour (45 minutes) with several educative activities which could be easily organized by a school teacher. Thus, this extra-curricular activity is potentially applied at schools. It is suggesting that the extra-curricular activity organizing as a seminar should be included in education activities at secondary and high schools to improve reproductive health knowledge for adolescents.

## Conclusion

The ethnic minority high school students’ knowledge of reproductive health was found to be relatively poor. Extra-curricular activities markedly increased the knowledge of many aspects of reproductive health. These findings suggest that it is necessary to improve the reproductive health knowledge for ethnic minority high school students in mountainous areas in Vietnam, and that extra-curricular activities organized as seminars are effective and suitable to provide and retain students’ knowledge of reproductive health.

***Statements: ***The present study has been approved by the Ethical Evaluation Committee in Biomedical Research and the Council of Ethics in Biomedical Research, the Ministry of Health, Vietnam and has been performed in accordance with the ethical standards as laid down in the 1964 Declaration of Helsinki and its later amendments or comparable ethical standards.

***Ethical approval:*** All procedures performed in this study involving human participants were in accordance with the ethical standards of the Ethical Evaluation Committee in Biomedical Research and the Council of Ethics in Biomedical Research, the Ministry of Health, Vietnam and with the 1964 Helsinki declaration and its later amendments or comparable ethical standards.
